# Dynamic Neuron-Glia Interactions in an Oscillatory Network Controlling Behavioral Plasticity in the Weakly Electric Fish, *Apteronotus leptorhynchus*

**DOI:** 10.3389/fphys.2017.01087

**Published:** 2017-12-22

**Authors:** Günther K. H. Zupanc

**Affiliations:** Laboratory of Neurobiology, Department of Biology, Northeastern University, Boston, MA, United States

**Keywords:** neural oscillator, neuron-glia interaction, plasticity, astrocytes, gliogenesis, structural reorganization, pacemaker nucleus, *Apteronotus leptorhynchus*

## Abstract

The involvement of glial cells in the regulation of physiological functions is being increasingly recognized, yet their role in plasticity of neural oscillators has remained largely elusive. An excellent model system to address the latter function is the pacemaker nucleus of the weakly electric fish, *Apteronotus leptorhynchus*. This brainstem oscillator drives the fish's electric organ discharge in a one-to-one fashion, with median frequencies of 880 Hz in males and 740 Hz in females. Morphometric analysis of the pacemaker nucleus has shown that astrocytes outnumber mature neurons seven-fold, and oscillator neurons even 200-fold. A similar dominance of astrocytes occurs among the adult-born cells that differentiate into glia and neurons. The astrocytes form a dense meshwork of cells interconnected by gap junctions. The degree of association of astrocytic fibers with the neural oscillator cells, and the gap-junction coupling between individual astrocytes, exhibit a sexual dimorphism, which parallels the sexual dimorphisms in the output frequency of the pacemaker nucleus, and ultimately in the electric organ discharge of the fish. It is hypothesized that the dynamics in astroglial structure mediate differences in the capacity to buffer potassium, which increases during the generation of action potentials. These differences, in turn, affect the excitability of the neural oscillator cells, and thus the output frequency of the pacemaker nucleus. Comparison of the pacemaker nucleus with other brain oscillators suggests that modulation of the output activity is one of the chief functions of the interaction of glia with the neural oscillator cells.

## Introduction

Since Maiken Nedergaard and Philip G. Haydon and associates reported in two independent papers in 1994 that stimulation of astrocytes results in elevated levels of Ca^2+^ in adjacent neurons (Nedergaard, 1994; Parpura et al., 1994), a plethora of studies have led to the notion that glial cells, particularly astrocytes, play an active role in the modulation of physiological functions of neural circuits. These studies have indicated that the modulatory actions of astrocytes can be grouped into two broad categories, according to the duration of their effects (Hatton, 1997; Theodosis et al., 2008; Haydon and Nedergaard, 2015). Transient changes, which operate on a second scale, are based on molecular signals exchanged between astrocytes and neurons. Long-term changes, on the other hand, occur over minutes, hours, or days and involve restructuring of glial morphology, in addition to molecular-signaling.

Despite the recognition of astrocytes as key players in brain plasticity, their role in functional plasticity of neural oscillators has received surprisingly little attention. A neural oscillator well characterized in terms of its behavioral function and the plasticity associated with the sexually dimorphic output pattern is the pacemaker nucleus in the medulla oblongata of the weakly electric fish *Apteronotus leptorhynchus* (Dye and Meyer, 1986). Although the existence of glia in this nucleus has been known since its early morphological description (Elekes and Szabo, 1985), their potential role in the regulation of the oscillatory activity has been examined only in recent years (Sîrbulescu et al., 2014; Zupanc et al., 2014). In the following, I will summarize the key findings, discuss them in the broader context of glia-neuron interactions in oscillatory networks, and propose a model to explain how astrocytes might modulate the activity of neurons in the pacemaker nucleus to produce a sexually dimorphic behavior.

## Cellular composition of the pacemaker nucleus

**Figure 1:**
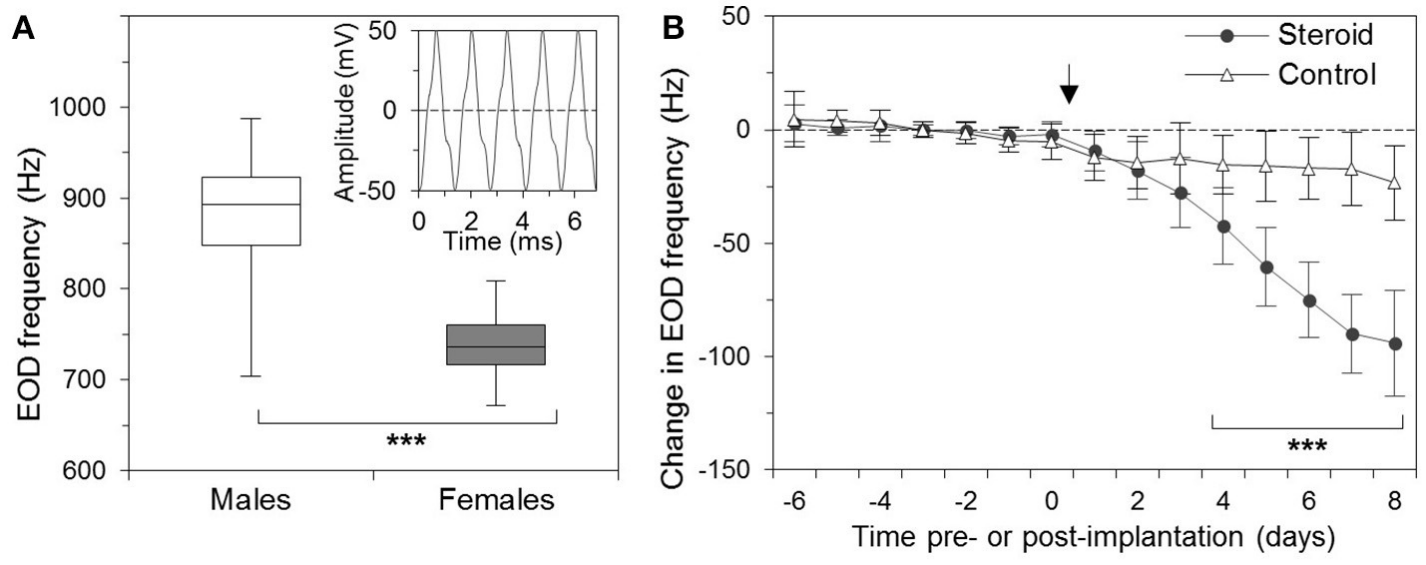
Sexual dimorphism of the electric organ discharge (EOD) of the weakly electric fish, *Apteronotus leptorhynchus*. **(A)** Box-and-whisker plot of the EOD frequencies of males (35) and females (48), adjusted to an ambient temperature of 26°C. Whiskers mark the minimum and maximum values in each group, and boxes indicate the interquartile ranges. Middle lines denote medians. The difference in the EOD frequency between males and females is highly significant (^***^). The inset shows a trace of the EOD signal. **(B)** Effect of β-estradiol on the EOD frequency of *Apteronotus leptorhynchus*. The EOD frequency was determined on 7 consecutive days before the operation (days −6 through 0) and on 8 days following the surgery (days 1 through 8). Fish received implants with (circles; *n* = 14) or without (triangles; *n* = 17) β-estradiol immediately following the measurement of the EOD frequency on day 0 (indicated by arrow). For each fish, the changes in EOD frequency were quantified relative to its average frequency over the 7-day pre-implantation period. The dashed line indicates no change from this baseline. Starting with day 4 after the implantation, the EOD frequencies of the β-estradiol-treated fish were significantly different from the discharge frequencies of the controls as well as from the pre-implantation frequencies (^***^) (From: Zupanc et al., 2014. Copyright permission not required due to author's own work).

Like all apteronotids, *A. leptorhynchus* possesses an electric organ formed by modified axons of spinal motor neurons, which are commonly referred to as electromotor neurons. The synchronous depolarization of these electrocytes results in electric organ discharges (EODs) in a species-specific frequency range of 650–1,000 Hz (de Oliveira-Castro, 1955; Bennett, 1971; Waxman et al., 1972). Within this frequency band, males discharge at higher frequencies than females (Figure 1A; Meyer et al., 1987; Zupanc et al., 2014).

The EOD frequency is controlled, in a one-to-one fashion, by the frequency of the oscillatory network defined by two of the neuronal cell types of the pacemaker nucleus, pacemaker cells and relay cells (Elekes and Szabo, 1985; Dye and Heiligenberg, 1987). While the pacemaker cells are restricted to the pacemaker nucleus, the relay cells project down the spinal cord to form electrotonic junctions with the electromotor neurons.

**Figure 2:**
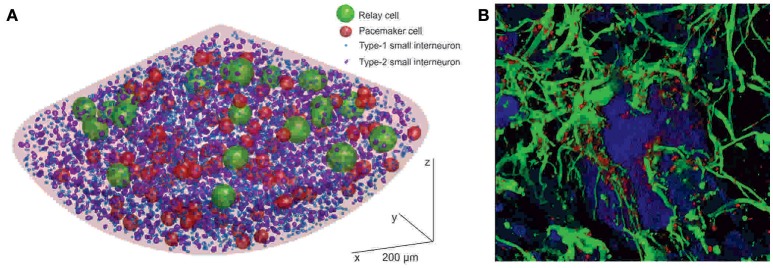
Cellular structure of the pacemaker nucleus of *Apteronotus leptorhynchus*. **(A)** Three-dimensional model of the neural organization of the pacemaker nucleus, based on a statistical-mapping approach. Neurons form three classes—relay cells, pacemaker cells, and small interneurons—distinguished by the sizes of their cell bodies. The small interneurons can be further divided into subtypes 1 and 2. Glial cells are not shown because, due to their abundance, they would cover essentially the entire area of the nucleus, making it impossible to visualize the neuronal cells in this representation. **(B)** Association of glia with neurons in the pacemaker nucleus. The image is based on a three-dimensional shadow projection of a z-stack of confocal images. Neurons expressing Hu C/D (blue) are embedded in a dense meshwork of GFAP-expressing astrocytes (green). The glia express the gap-junction protein connexin-43 in a punctate pattern (red). In the center of the image, a pacemaker cell (P), surrounded by a dense astrocytic syncytium, is shown. Scale bar = 5 μm (After: Sîrbulescu et al., 2014. Copyright permission not required due to author's own work).

Although pacemaker and relay cells are the only neuronal cells known to be involved in the generation of the oscillatory activity of the pacemaker nucleus, they are—with 87 and 20, respectively—a diminishing minority among neurons. The vast majority of neurons (all of which have been identified by their immunoreactivity against the neuron-specific marker protein Hu C/D) are small interneurons (Figure 2A; Elekes and Szabo, 1985; Turner and Moroz, 1995; Zupanc et al., 2014). They total ~5,300 in mid-aged adult fish (Elekes and Szabo, 1985; Turner and Moroz, 1995; Sîrbulescu et al., 2014; Zupanc et al., 2014). Nevertheless, even when considering all three types of neurons combined, they comprise only a minute fraction of the roughly 80,000 cells whose cellular identity is known. (The pacemaker nucleus of a mid-aged fish is formed by an estimated 200,000 cells; the identity of ~120,000 cells remains unknown). Besides neurons, stem/progenitor cells (~35,000; see section “Development of Astrocytes and Neurons in the Adult Pacemaker Nucleus,” below) and glia (~40,000; Sîrbulescu et al., 2014) have been identified. The latter express a variety of glial marker proteins, including S100, glial fibrillary acidic protein (GFAP), glutamine synthetase, and vimentin, with a large degree of overlap among the individual expression patterns.

The above morphometric data indicate that, among identified cells, glia outnumber the three types of neurons more than 7-fold, and the pacemaker and relay cells even more than 200-fold. A glia-neuron ratio of 7:1, as found among identified cells in the pacemaker nucleus, is, by any means, extremely high. For comparison, in the spinal cord of *A. leptorhynchus* S100-expressing glia and Hu C/D-expressing neurons coexist in a 2:1 ratio (Sîrbulescu et al., 2017). Importantly, these two ratios are directly comparable, as they have been determined in the pacemaker nucleus and the spinal cord by employing identical immunostaining and image processing protocols. On the other hand, the 2:1 ratio in the spinal cord of *A. leptorhynchus* is in line with measures in various regions of rat and primate brains, which yield typical glia-to-neuron ratios between 0.5 and 2 (Hilgetag and Barbas, 2009; von Bartheld et al., 2016).

## Development of astrocytes and neurons in the adult pacemaker nucleus

Morphometric analysis of the pacemaker nucleus of fish of various ages has demonstrated an age-related increase in the total number of cells during adulthood, from ~100,000 cells in young adults to 300,000 in adults toward the end of their lives (Sîrbulescu et al., 2014). However, whereas the small interneuron population grows at the same rate as the total number of pacemaker nucleus cells, no age-dependent increase in cell numbers has been found among pacemaker and relay cells—their numbers are remarkably constant over adult life.

The continuous growth of the pacemaker nucleus during adulthood is the result of continuous generation of new neurons and glial cells from adult stem/progenitor cells, most of which express the stem-cell markers Sox2 and Meis 1/2/3 (Sîrbulescu et al., 2014). However, only a minor fraction (2–3%) of them are mitotically active, whereas the rest are quiescent at any given time point. Further characterization of the stem/progenitor cells has shown that the vast majority of them express S100 and assume morphological properties of radial glia. Thus, these observations suggest that radial glia-like precursors that express Sox2 and Meis 1/2/3 serve as the adult stem/progenitor cells, which give rise to the new neurons and glia in the pacemaker nucleus. Radial glia have also been identified as endogenous multipotent stem cells in the adult mammalian brain (Doetsch et al., 1999; Laywell et al., 2000; Seri et al., 2001; Bonaguidi et al., 2011).

Combination of labeling of S-phase cells with a single pulse of the thymidine analog 5-bromo-2′-deoxyuridine, and immunostaining against the neuron-specific marker protein Hu C/D and the glia-specific marker protein S100 after various chase times, has revealed some notable details of the developmental pattern of the adult-born cells in the pacemaker nucleus (Sîrbulescu et al., 2014). At early stages of the cellular development, about equal portions of the progeny acquire neuronal and astrocytic identities. However, starting at about 10 days, the number of young neurons decreases, while the number of cells that differentiate into astrocytes continuously increases, resulting, at 100 days of cellular age, in a glia-to-neuron ratio of 4:1. Such a ratio is in line with the dominance of astrocytes observed among the entire population of mature cells in the pacemaker nucleus.

## Glial dynamics in the pacemaker nucleus associated with behavioral plasticity

The abundance of astrocytes, relative to the number of neurons, and in particular to the number of pacemaker and relay cells, prompts the question of what role glia play in the pacemaker nucleus. Some important insights toward finding an answer to this question have been provided by analysis of the structural interconnection of pacemaker and relay cells with astrocytes, and the dynamics of this system. Three-dimensional reconstruction of pacemaker nucleus tissue based on confocal laser scanning micrographs has revealed a dense meshwork of astrocytic fibers closely associated with the pacemaker and relay cells (Figure 2B; Zupanc et al., 2014), reminiscent of the close apposition of glial processes to pacemaker and relay cells revealed by electron microscopy (Elekes and Szabo, 1985). These S100-immunopositive astrocytes express high levels of connexin-43, a member of the connexin family of transmembrane gap junction proteins that plays a critical role in the formation of the astrocytic syncytium (Giaume and Liu, 2012). Thus, the morphological data suggest that the pacemaker and relay cells are embedded in a dense meshwork of astrocytes interconnected by gap junctions to form an astrocytic syncytium.

The astrocytic syncytium undergoes dynamic changes in concert with alterations in the EOD frequency. As mentioned above, males and females occupy different frequency domains within the species-specific EOD range. At a water temperature of 26°C, the mean EOD frequency of males is ~880 Hz, whereas the mean frequency of females is ~740 Hz (Figure 1A). This sexual dimorphism is controlled by steroid hormones. Chronic administration of β-estradiol results in both males and females in a gradual decrease of the EOD frequency (Figure 1B; Meyer et al., 1987; Schaefer and Zakon, 1996; Zupanc et al., 2014).

The sexual dimorphism in the fish's EOD frequency is paralleled by changes in the morphology and expression pattern of the astrocytes in the pacemaker nucleus (Zupanc et al., 2014). In females, the pacemaker nucleus is occupied by numerous, intensely labeled GFAP-immunoreactive fibers. In males, the number and intensity of labeling of such fibers is significantly lower. This overall difference extends to individual pacemaker and relay cells. Quantitative analysis has shown that the total GFAP labeling associated with the area covered by pacemaker and relay cells is significantly higher in females than in males. Similarly, the relative area covered by connexin-43 immunoreactivity in the region around pacemaker cells is almost 3-times larger in females than in males.

The changes in astrocytic architecture that occur as part of the development of the sexual dimorphism in the structure and function of the pacemaker nucleus could be due to the generation of new astrocytes, or the outgrowth of existing astrocytes, or both, and are probably controlled by estradiol. Evidence in support of both estradiol-induced gliogenesis and astrocytic remodeling has been obtained in mammals. In mice, sex differences have been demonstrated in cell-cycle regulation of hematopoietic stem cells. Such cells divide significantly more frequently in females than in males—an effect that can be mimicked by administration of estradiol in both males and females (Nakada et al., 2014). In the basal hypothalamus of mammals, the portion of neuronal somata covered by astrocytic processes fluctuates over the estrous cycle, being high when estrogen levels are elevated, and low when estrogen levels have decreased (García-Segura et al., 1994). The retraction and elongation of astrocytic processes over the surfaces of neurons can be mimicked by administration of estradiol to ovariectomized animals, an effect that occurs as rapidly as within 2 h after the steroid hormone application (Langle et al., 2003). It is likely that this modulatory effect of estradiol on glial structure is direct, as estrogen receptors have been found in astrocytic cells of hypothalamic areas (Langub and Watson, 1992).

## What is the function of astrocytes in the pacemaker nucleus?

The relative abundance of astrocytes, the dominance of gliogenesis over neurogenesis, the close association of astrocytic processes with pacemaker and relay cells, the structural reorganization of the astrocytic syncytium occurring in concert with sexual dimorphism-related changes in the oscillatory output frequency of the pacemaker nucleus—taken together, these observations point to a role of astrocytes in mediating the function of the pacemaker nucleus as the master oscillator in the neural network that controls the EOD frequency. However, despite this compelling correlative evidence, the role of the astrocytic meshwork in the regulation of the pacemaker frequency, and thus the EOD frequency, has remained elusive.

Given the high-frequency firing of the pacemaker and relay cells and the resulting continuous release of potassium from these cells into the extracellular space, it is plausible to hypothesize that one of the chief functions of the astrocytic meshwork in the pacemaker nucleus is to buffer the increases in K^+^ concentrations. Potassium buffering by glia was first suggested over half a century ago (Hertz, 1965). Since then, a large body of experimental evidence has established astrocytic buffering of excess K^+^ as a key concept of glial function in the nervous system (Kofuji and Newman, 2004). It is thought that the uptake of extracellular K^+^ is primarily achieved via Na^+^/K^+^-ATPase, but K^+^ channels have also been implicated in this function. It is, therefore, worth noting that Kv1.2 potassium channels have been identified through immunohistochemical staining in thin fibers resembling the astrocytic processes that wrap around pacemaker and relay cells, but are absent from these neurons (Smith and Zakon, 2000). Following uptake of extracellular K^+^, the abundant coupling of astrocytes via gap junctions, as indicated by the intense connexin-43 immunoreactivity associated with astrocytic cells (Zupanc et al., 2014), might serve as a mechanism to aid redistribution of K^+^ from sites of excessive levels to sites of lower K^+^ concentrations within the pacemaker nucleus.

The astrocytic syncytium surrounding the pacemaker and relay cells may be involved not only in elimination of excess K^+^ ions from the extracellular space, but also in regulating the output frequency of the pacemaker because a major determinant of the membrane potential is the extracellular K^+^ concentration. An increase in the extracellular K^+^ concentration leads to a reduction in the K^+^ equilibrium potential, whereas a decrease in the extracellular K^+^ concentration results in an increase in the K^+^ equilibrium potential. In either case, these changes affect the excitability of neurons by altering the difference between the resting potential and the threshold potential at which voltage-gated sodium channels are activated (Wang et al., 2012).

Some important insights into the role of astrocytic buffering of K^+^ in the generation of high-frequency oscillations has been gained by studying epilepsy and experimental models of this condition. Epileptic seizures are characterized by high-frequency oscillations (Bragin et al., 1999; Jirsch et al., 2006; Pearce et al., 2013). Impairment of K^+^ buffering induced in astrocytes by altering the expression of inwardly rectifying K^+^ channels enhances the susceptibility for epileptic seizures (Steinhäuser et al., 2012). Similarly, seizures can also be triggered by increases in the extracellular K^+^ concentration (Traynelis and Dingledine, 1988; Rangroo Thrane et al., 2013).

Conversely, enhancement of the efficiency in K^+^ uptake and inter-astrocytic redistribution will reduce the excitability of neural networks by widening the gap between the resting membrane potential and the threshold potential at which action potentials are triggered. Such increase in astrocytic buffering capacity is predicted to result in a reduction in output frequency of neural oscillators. In the pacemaker nucleus, enhanced buffering capacity might be achieved by increasing the surface area of pacemaker and relay cells covered by astrocytic processes, and by strengthening gap junction coupling between individual astrocytes.

## Model of the development of the sexually dimorphic EOD frequency

Based on the above empirical evidence and theoretical considerations, the following working hypothesis emerges to mechanistically explain the development of the sexual dimorphism in EOD frequency of *A. leptorhynchus*: Differences in the association of the astrocytic syncytium with pacemaker and relay cells in the pacemaker nucleus develop as part of sexual differentiation under the direct control of steroid hormones, foremost estradiol. In females, the larger surface area of pacemaker and relay cells covered by astrocytic processes, accompanied by increases in specific types of K^+^ channel and/or in Na^+^/K^+^-ATPase expression, and the stronger gap-junction coupling of the individual astrocytes within the astrocytic syncytium, serve as mechanisms to increase astrocytic K^+^ buffering. As a result, the levels of extracellular K^+^ are reduced. This reduction leads to a decrease in the excitability of the pacemaker and relay cells, thereby lowering the oscillator frequency of the pacemaker nucleus, and ultimately the EOD frequency of the fish.

## Conclusions

Involvement of glial cells in neural oscillations has been suspected since circadian fluctuations in GFAP labeling in the suprachiasmatic nucleus of hamsters (Lavialle and Servière, 1993; Lavialle et al., 2001) and in the abundance of clock gene products in glial cells of *Drosophila* (Siwicki et al., 1988; Zerr et al., 1990; Ewer et al., 1992) were first reported. Despite accumulating (mostly circumstantial) evidence, the precise role of glia in the regulation of oscillatory activity has remained largely a matter of speculation. A key question is whether glia in different oscillatory networks share similar functions and mechanisms that mediate these functions, despite major differences in cellular organization and output patterns. Besides the pacemaker nucleus of *A. leptorhynchus*, two other oscillatory systems in which the role of glia has been studied are circadian clocks and olfactory glomeruli. While the period of the pacemaker nucleus oscillations is on the order of 1 ms, corresponding values of circadian rhythms approximate 1 day. Mitral cells in individual olfactory glomeruli of mice exhibit oscillations in membrane potential with period lengths somewhat in between these two extremes, namely ~5 s (Roux et al., 2015).

Notwithstanding the diversity of these systems, functional studies support the notion that the role of glia in these oscillatory networks is similar. Manipulation of clock genes specifically in astrocytes within the suprachiasmatic nucleus of mice has indicated that the astrocytic circadian clock, although neither necessary nor sufficient for sustaining circadian rhythms, interacts with the neuronal clock system to modulate the period length, and thus the frequency, of the circadian rhythm (Tso et al., 2017). Similarly, disruption by toxins of the metabolism of glial cells in the visual clock system of dipterans does not abolish circadian rhythms but affects the amplitude of circadian fluctuations in the size of neurons within this system (Pyza and Górska-Andrzejak, 2004). Lastly, combination of genetic and pharmacological approaches in the olfactory bulb system has shown that the activity of gap-junction channels interconnecting astrocytes modulates the amplitude and the firing rate of mitral cells (Roux et al., 2015).

Taken together, structural and functional studies suggest that astrocytes, although not essential for the generation of neural activity in oscillatory networks, play a critical role in the modulation of the output pattern of such networks. They appear to exert this function by undergoing both short-term and long-term plastic changes in morphological structure, ion channel expression, and/or gap-junction coupling between individual astrocytic cells. Although many details of the modulatory action of glia remain to be elucidated, it becomes increasingly clear that biologically realistic models of plasticity of oscillatory neural networks will have to take the role of glia-neuron interactions in this phenomenon into account.

## Author contributions

The author confirms being the sole contributor of this work and approved it for publication.

### Conflict of interest statement

The author declares that the research was conducted in the absence of any commercial or financial relationships that could be construed as a potential conflict of interest.
